# Parametric Analysis of Free Vibration of Functionally Graded Porous Sandwich Rectangular Plates Resting on Elastic Foundation

**DOI:** 10.3390/ma17102398

**Published:** 2024-05-16

**Authors:** Bin Qin, Jie Mei, Qingshan Wang

**Affiliations:** 1 Key Laboratory of Traffic Safety on Track, Ministry of Education, School of Traffic & Transportation Engineering, Central South University, Changsha 410075, China; qinbin@csu.edu.cn (B.Q.); jiemei@csu.edu.cn (J.M.); 2 Joint International Research Laboratory of Key Technology for Rail Traffic Safety, Central South University, Changsha 410075, China; 3 National & Local Joint Engineering Research Center of Safety Technology for Rail Vehicle, Central South University, Changsha 410075, China; 4 State Key Laboratory of High Performance Complex Manufacturing, Central South University, Changsha 410083, China

**Keywords:** functionally graded porous sandwich rectangular plates, parametric analysis, free vibration, elastic foundation, unified solution

## Abstract

Based on the three-dimensional elasticity theory, the free vibration of functionally graded porous (FGP) sandwich rectangular plates is studied, and a unified solution for free vibration of the plates is proposed in this study. The arbitrary boundary conditions of FGP sandwich rectangular plates are simulated by using the Rayleigh–Ritz method combined with artificial spring theory. The calculation performances of the unified solution for FGP sandwich rectangular plates such as convergence speed and computational efficiency are compared extensively under different displacement functions. In addition, three kinds of elastic foundation (Winkler/Pasternak/Kerr foundations) and three porosity distributions are considered. Some benchmark results and accurate values for the free vibration of FGP sandwich rectangular plates resting on elastic foundations are given. Finally, the effects of diverse structural parameters, elastic foundations with different parameters, and boundary conditions on the free vibration of the FGP sandwich rectangular plates are analyzed.

## 1. Introduction

The sandwich structure exceeds ordinary single materials in terms of insulation and noise reduction, especially in terms of the mechanical property [1,2], which is generally made of various materials, such as aluminum alloy, stainless steel, and nonmetal materials (like ceramic) [3,4]. The sandwich plate with an aluminum/ceramic structure has extensive applications in the transportation, architectural, and aerospace industries for its characteristics of high tensile strength, hardness, and compressive strength, which can give full play to the advantages of composite materials. Porous metal has also attracted popularity for its light weight, high-energy absorption, and controllable permeability [5]. The components of the functionally gradient material show the characteristics of gradually changing (linear, non-linear, or specific functions) in space, which can achieve a variety of different performances inside the material, with excellent efficiency under different stress and environmental conditions [6]. Furthermore, alumina ceramic material is proved to have the advantages of high strength, corrosion resistance, and good thermal stability, which can be an excellent reinforcement for developing composite materials [7,8]. By combining the sandwich structure, porous metal, functional gradient material, and alumina ceramics, FGP sandwich-reinforced composites can be obtained, which may have the advantages of all these materials.

In previous research, many studies have been carried out for the vibration and curvature characteristics of functionally gradient porous sandwich materials, which involve different types of structure, such as linear and curved beams [9] as well as rectangular and circular plates [10,11]. Among these structures, plates are frequently encountered in various industrial fields. Li et al. [12,13] proposed several novel theories to investigate the effect of some key geometric parameters and material properties on the deflections, stresses, and static responses of functionally graded plates. By adopting the refined first-order shear deformation theory and the equations of motion derived by Lagrange equations, Bathini et al. [14] studied the free vibration of bidirectional FGP plates. Concerning the free vibration response of functionally graded plates, Merdaci et al. [15] used the higher-order shear deformation plate theory to deduce the formulations for parameter research. The static bending and free vibration of functionally graded plates with random porosity were analyzed by Sun et al. [16], based on the novel systematic spectral stochastic isogeometric analysis. The effects of the key parameters of an in-plane bidirectional FGP rectangular plate on its nonlinear frequency were investigated based on the Hamilton principle and von Karman nonlinearity strain–displacement relations by Hashemi et al. [17]. A novel semi-analytical plate formulation was proposed by Zang et al. [18] to investigate the effects of the gradient index and aspect ratios on the static and free vibration responses of FGP plates. The effects of the key parameters on the bending and free vibration of the functionally graded plates were investigated by Hadji and Alazwari et al. [19,20], which revealed the potential effects of geometric parameters and material properties on the vibration characteristics and mechanical properties of the plates. Actually, rectangular plates can be placed on the soil medium and can be regarded as resting on an elastic foundation. Therefore, the influence of the interaction between the rectangular plate and the elastic foundation on the vibration performance of the rectangular plate has attracted much attention. Therefore, the effects of the interaction between rectangular plates and elastic foundations on vibration are worth studying, which is a pretty popular research area now. The Winkler model is a simple elastic foundation model, which consists of several uniform linear springs arranged around the outer surface of a rectangular plate. Through an additional shear layer, the Pasternak foundation can be gained. Various investigations on rectangular plates with rectangular plates embedded in elastic foundations can be found in the literature [21]. Further, the Kerr foundation with three parameters has also been proposed, which consists of two elastic layers and a shear layer [22].

For the study of sandwich rectangular plates, the Rayleigh–Ritz method on vibration analysis has been significantly improved and optimized. Jing et al. [23] proposed a variable stiffness optimization algorithm based on a layer-wise optimization approach and first-order shear deformation theory, which can greatly improve the buckling resistance capacity of variable-angle tow plates. Belardi et al. [24] discretized the displacement field of composite sector plates with rectilinear orthotropy into linear combinations of several approximate functions according to classical lamination theory and the Ritz method. For the Rayleigh–Ritz method, the key factor for the high performance of solutions is to choose an appropriate admissible displacement function. Various polynomials are used to represent the admissible displacement function (Chebyshev [25], Legendre [26], orthogonal [27], modified Fourier series [28], and Fourier–Bessel polynomials [29]). Huo et al. [25] employed the Chebyshev polynomial and Fourier series to decompose the unified solution of the stress function to analyze the transverse vibration and buckling characteristics of the rectangular plate via the Ritz method. Kumar [27] studied the free transverse vibration of functionally graded rectangular plates with porosity effects under simply supported conditions based on classical plate theory, using the boundary characteristic orthogonal polynomials. Yang et al. [30] derived the formulations of viscoelastic and functionally graded sandwich plates under arbitrary boundary conditions through the linear superposition of a double Fourier series and auxiliary functions and the Rayleigh–Ritz method. Modified Fourier series and Fourier–Bessel polynomials were also used to assess the vibration behavior of functionally gradient plates on elastic foundations, overcoming the discontinuity problem in the solution process by adding additional terms [31,32]. In general, the corresponding formulation for vibration analysis will change with different admissible displacement functions, so the computational performance with different admissible displacement functions is worth studying.

All in all, the previous research on FGP sandwich rectangular plates may have the following defects or aspects that have not been considered: (1) the more accurate three-dimensional solution of the FGP sandwich rectangular plates has not yet been carried out; (2) parametric research (including structural parameters and material properties) is insufficient; (3) the influence between the vibration behavior of FGP sandwich rectangular plates and elastic foundations has not been fully studied. The purpose of this study is to put forward a unified solution for solving the vibration problems of FGP sandwich rectangular plates resting on elastic foundations. Winkler, Pasternak, and Kerr foundations are taken into consideration, and the Rayleigh–Ritz method is adopted to deduce formulations of the three-dimensional elastic theory. Arbitrary boundary conditions should be considered and studied through the artificial spring technique. Various polynomials are taken to express the admissible displacement functions, and their influence degree on the results is compared in detail. As for the material properties, several geometric parameters, porosity distributions, thickness ratios, porosity coefficients, and weight fractions are also considered. Finally, some new results are presented, and a parametric study is performed to fully explain the free vibration of FGP sandwich rectangular plates.

## 2. Basic Formulations

### 2.1. Description of Sandwich Plates Resting on Elastic Foundations

**Figure 1:**
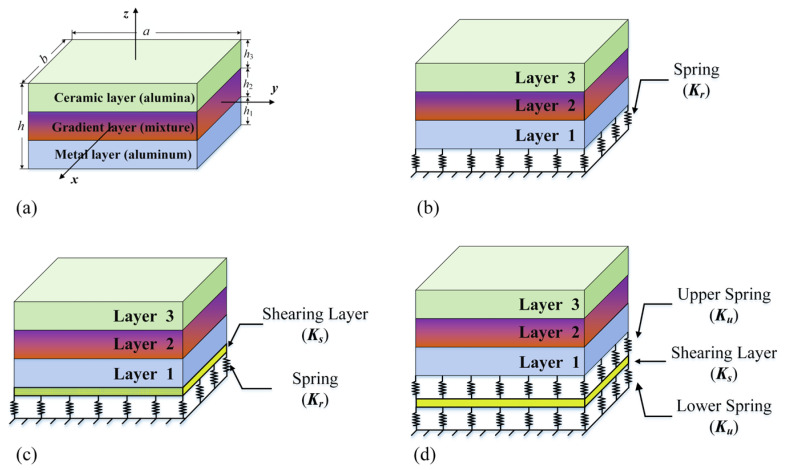
(**a**) Coordinates and geometry of an FGP sandwich rectangular plate and a main view of (**b**) Winkler, (**c**) Pasternak, (**d**) Kerr foundation.

As shown in Figure 1a, a rectangular plate composed of a functionally gradient material is presented, with a length of *a*, width *b,* and thickness *h*. The top layer of the functionally graded sandwich rectangular plate is ceramic, the bottom layer is aluminum, and the sandwich layer is composed of mixed-gradient material. Taking a three-dimensional Cartesian coordinate system as the reference, the coordinate system is located at the geometric center of the rectangular plate, where *x*, *y*, and *z* are along the length, width, and thickness directions, respectively. Then, the arbitrary position of the rectangular plate can be expressed within a range between *–h*/2 and *h*/2 in the thickness direction. The bottom surface of the metal layer of the rectangular plate is continuously surrounded by an elastic foundation, which contains Winkler (Figure 1b), Pasternak (Figure 1c), and Kerr foundations (Figure 1d). For the Winkler foundation, the distributed load is uniform in both the horizontal and vertical directions, which is achieved using several linear springs with stiffness *K_r_* uniformly distributed, and each spring represents a ground point. For the Pasternak foundation, considering the horizontal and shear stiffness of the foundation, it uses an elastic model, which contains *K_r_* and *K_g_* (a stiffness parameter on the shear layer) to simulate vertical and shear spring reactions. The Kerr foundation can be replaced by an elastic model with three parameters, where the outer surface is surrounded by lower springs of stiffness *K_l_*, shear layer of stiffness *K_s_*, and upper springs of stiffness *K_u_*. By adding linear springs to describe the linear characteristics, the complex behavior of the foundation can be accurately simulated [22]. It is worth considering that, although the Kerr foundation has three parameters, the Kerr and Pasternak foundations have the same mathematical concepts. The three different foundations can be transformed into each other under certain condition while they have different physical meanings. By setting *K_g_* = 0, the Pasternak foundation can be transformed into the Winkler model. Moreover, the Kerr foundation is a generalization of the Pasternak model [33].

### 2.2. Description of FGP Materials

The material properties of FGP sandwich rectangular plates vary continuously due to the gradually changing volume fraction of various components (ceramic and metal), usually in the thickness direction only. The power-law function is commonly used to describe the expression of stiffness and density of functionally gradient materials along the thickness direction [34], which can be expressed as:

(1) $$\begin{array}{l} Vf_{1}=0,\;z\in [\frac{-h_{1}-h_{2}-h_{3}}{2},\frac{h_{1}-h_{2}-h_{3}}{2}]\\ Vf_{2}={\left(\frac{z-h_{2}}{h_{3}-h_{2}}\right)}^{k},\;z\in [\frac{h_{1}-h_{2}-h_{3}}{2},\frac{h_{1}+h_{2}-h_{3}}{2}]\\ Vf_{3}=1,\;z\in [\frac{h_{1}+h_{2}-h_{3}}{2},\frac{h_{1}+h_{2}+h_{3}}{2}] \end{array}$$

where *Vf_i_* (*i* = 1, 2, 3) denotes the volume fraction function of the *i*th layer, and *k* is the volume fraction index (0 ≤ *k* < ∞), which dictates the material variation degree in the thickness direction.

The upper and bottom layers of sandwich rectangular plates are made of ceramic and metal, respectively, while the cores of the sandwich rectangular plates are of three types of functionally graded material, as mentioned before, whose mathematical forms are discussed as follows [22]:

(1) Porosity distribution *P*_1_:

Layer 1 and Layer 3 of the sandwich rectangular plate are composed of metal and ceramic (as shown in Figure 1), so the material parameters of Layer 1 and Layer 3 are the same as metal and ceramic, respectively. The gradient layer in the middle changes continuously in the thickness direction with the material composition (ceramic and metal), and the pores are evenly distributed in it. Considering the pore structure, the Young’s modulus and density of the gradient layer are jointly determined by the proportion of metal, ceramic, and air (the density and Young’s modulus of air can be ignored compared with the other two materials). Thus, the material parameters of the FGP sandwich plate can be expressed as:

(2) $$\begin{array}{l} E_{1}^{1}=E_{m},\;\rho_{1}^{1}=\rho_{m}\\ E_{2}^{1}=Vf_{2}\cdot E_{c}+(1-Vf_{2})E_{m}-\frac{e_{0}}{2}(E_{c}+E_{m})\\ \rho_{2}^{1}=Vf_{2}\cdot \rho_{c}+(1-Vf_{2})\rho_{m}-\frac{e_{0}}{2}(\rho_{c}+\rho_{m})\\ E_{3}^{1}=E_{c},\;\rho_{3}^{1}=\rho_{c} \end{array}$$

where $E_{i}^{j}$ and $\rho_{i}^{j}$ represent the Young’s modulus and density of the material within the *i*th layer with *P_j_* porosity distribution. *E_m_*, *E_c_*, *ρ_m_* and *ρ_c_* denote the Young’s modulus and density of metal and ceramic materials, respectively, and their value will be explained later. The porosity coefficient is *e*_0_.

(2) Porosity distribution *P*_2_:

Based on porosity distribution *P*_1_, considering a situation in which the porosity may be unevenly distributed, the parameter *Vp* is introduced, which can indicate the symmetrical pattern of the porosity distribution. Then, the material parameters of the FGP sandwich plate can be indicated:

(3) $$\begin{array}{l} E_{1}^{2}=E_{m},\;\rho_{1}^{2}=\rho_{m}\\ Vp=1-\frac{\left|2z-h_{2}-h_{3}\right|}{h_{3}-h_{2}}\\ E_{2}^{2}=Vf_{2}\cdot E_{c}+(1-Vf_{2})E_{m}-\frac{e_{0}}{2}(E_{c}+E_{m})Vp\\ \rho_{2}^{2}=Vf_{2}\cdot \rho_{c}+(1-Vf_{2})\rho_{m}-\frac{e_{0}}{2}(\rho_{c}+\rho_{m})Vp\\ E_{3}^{2}=E_{c},\;\rho_{3}^{2}=\rho_{c} \end{array}$$

(3) Porosity distribution *P*_3_:

Based on porosity distribution *P*_2_, a modified porosity coefficient *Vk* is introduced, which can describe an uneven model expanded with a logarithmic function. And the material parameters of the FGP sandwich plate under this model can be expressed as:

(4) $$\begin{array}{l} E_{1}^{3}=E_{m},\;\rho_{1}^{3}=\rho_{m}\\ Vp=1-\frac{\left|2z-h_{2}-h_{3}\right|}{h_{3}-h_{2}},\;Vk=\ln(1+\frac{e_{0}}{2})\\ E_{2}^{3}=Vf_{2}\cdot E_{c}+(1-Vf)E_{m}-Vk(E_{c}+E_{m})Vp\\ \rho_{2}^{3}=Vf_{2}\rho_{c}+(1-Vf)\rho_{m}-Vk(\rho_{c}+\rho_{m})Vp\\ E_{3}^{3}=E_{c},\;\rho_{3}^{2}=\rho_{c} \end{array}$$

### 2.3. Kinematic Relations and Energy Expressions

According to the three-dimensional plate theory of elasticity, the relationships between strain and displacement can be described as:

(5) $$\begin{array}{ccc} \varepsilon_{x}=\frac{\partial u}{\partial x} & \varepsilon_{y}=\frac{\partial v}{\partial y} & \varepsilon_{z}=\frac{\partial w}{\partial z}\\ \gamma_{xy}=\frac{\partial u}{\partial y}+\frac{\partial v}{\partial x} & \gamma_{xz}=\frac{\partial u}{\partial z}+\frac{\partial w}{\partial x} & \gamma_{yz}=\frac{\partial v}{\partial z}+\frac{\partial w}{\partial y} \end{array}$$

where *u*, *v* and *w* represent the displacement components in the *x*, *y* and *z* directions, respectively; $\varepsilon_{x},\;\varepsilon_{y}$ and $\varepsilon_{z}$ denote the normal strains; $\gamma_{xy},\;\gamma_{xz}$ and $\gamma_{yz}$ signify the shear strains. Following the Hooke’s law, the universal relations of stress and strain can be expressed as:

(6) $$\left\{\begin{array}{c} \sigma_{x}\\ \sigma_{y}\\ \sigma_{z}\\ \tau_{xy}\\ \tau_{xz}\\ \tau_{yz} \end{array}\right\}=\left[\begin{array}{cccccc} Q_{11} & Q_{12} & Q_{13} & 0 & 0 & 0\\ Q_{12} & Q_{22} & Q_{23} & 0 & 0 & 0\\ Q_{13} & Q_{23} & Q_{33} & 0 & 0 & 0\\ 0 & 0 & 0 & Q_{44} & 0 & 0\\ 0 & 0 & 0 & 0 & Q_{55} & 0\\ 0 & 0 & 0 & 0 & 0 & Q_{66} \end{array}\right]\left\{\begin{array}{c} \varepsilon_{x}\\ \varepsilon_{y}\\ \varepsilon_{z}\\ \gamma_{xy}\\ \gamma_{xz}\\ \gamma_{yz} \end{array}\right\}$$

where $\sigma_{x},\;\sigma_{y}$ and $\sigma_{z}$ express normal stresses; $\tau_{xy},\;\tau_{xz}$ and $\tau_{yz}$ signify the shear stresses; $Q_{ij}\;\;(i,\;j=1\sim 6)$ expresses the elastic constants and can be written as:

(7) $$\left\{\begin{array}{l} Q_{11}=Q_{22}=Q_{33}=\frac{E(1-\mu )}{\left(1+\mu )(1-2\mu \right)}\\ Q_{12}=Q_{13}=Q_{23}=\frac{E\mu }{\left(1+\mu )(1-2\mu \right)}\\ Q_{44}=Q_{55}=Q_{66}=\frac{E}{2(1+\mu )} \end{array}\right.$$

The strain energy $U_{s}$ of the FGP sandwich rectangular plates can be expressed in light of the kinematic relations as:

(8) $$\begin{array}{l} U_{s}=\frac{1}{2}\int_{V}(\sigma_{x}\varepsilon_{x}+\sigma_{y}\varepsilon_{y}+\sigma_{z}\varepsilon_{z}+\tau_{xy}\gamma_{xy}+\tau_{xz}\gamma_{xz}+\tau_{yz}\gamma_{yz})dV\\ \;\;\;\;\;=\frac{1}{2}\int_{V}\left\{\begin{array}{l} Q_{11}{\left(\frac{\partial u}{\partial x}\right)}^{2}+2Q_{12}\frac{\partial u}{\partial x}\frac{\partial v}{\partial y}+2Q_{13}\frac{\partial u}{\partial x}\frac{\partial w}{\partial z}\\ +2Q_{23}\frac{\partial v}{\partial y}\frac{\partial w}{\partial z}+Q_{22}{\left(\frac{\partial v}{\partial y}\right)}^{2}+Q_{33}{\left(\frac{\partial w}{\partial z}\right)}^{2}\\ +Q_{44}{\left(\frac{\partial u}{\partial y}+\frac{\partial v}{\partial x}\right)}^{2}+Q_{55}{\left(\frac{\partial u}{\partial z}+\frac{\partial w}{\partial x}\right)}^{2}+Q_{66}{\left(\frac{\partial v}{\partial z}+\frac{\partial w}{\partial y}\right)}^{2} \end{array}\right\}dV \end{array}$$

In this study, a set of continuously distributed boundary springs are employed to simulate the boundary conditions [35]. At edge *x* = −*a*/2 (or edge *x* = *a*/2), six groups of linear springs with stiffness $k_{x0}^{u},\;k_{x0}^{v},\;k_{x0}^{w},\;k_{y0}^{u},\;k_{y0}^{v}$ and $k_{y1}^{w}$ (or $k_{x1}^{u},\;k_{x1}^{v},\;k_{x1}^{w},\;k_{y1}^{u},\;k_{y1}^{v}$ and $k_{y1}^{w}$) are introduced, where the subscript *x*0 and *x*1 represent the left boundary (*x* = −*a*/2) and right boundary (*x* = *a*/2) in the *x* direction (*y* direction is the same as the *x* direction). By changing the values of each stiffness, we could simulate different boundary conditions. The boundary conditions of FGP sandwich plates are imposed on four sides of the plate. In the process of the quadrature of the boundary potential energy of FGP sandwich plates, for example, on the boundary *x* = 0, which is the integral of potential energy of the *yoz* plane in Figure 1a, the potential energy $U_{bc}$ retained from the boundary springs can be expressed as:

(9) $$\begin{array}{l} U_{bc}=\frac{1}{2}\int_{-h/2}^{h/2}\int_{-b/2}^{b/2}[(k_{x0}^{u}+k_{x1}^{u})u^{2}+(k_{x0}^{v}+k_{x1}^{v})v^{2}+(k_{x0}^{w}+k_{x1}^{w})w^{2}]dydz\\ \;\;\;\;\;\;+\frac{1}{2}\int_{-h/2}^{h/2}\int_{-a/2}^{a/2}[(k_{y0}^{u}+k_{y1}^{u})u^{2}+(k_{y0}^{v}+k_{y1}^{v})v^{2}+(k_{y0}^{w}+k_{y1}^{w})w^{2}]dxdz \end{array}$$

*T* represents the kinetic energy and can be signified as:

(10) $$T=\frac{1}{2}\int_{-h/2}^{h/2}\int_{-b/2}^{b/2}\int_{-a/2}^{a/2}\rho [{\left(\frac{\partial u}{\partial t}\right)}^{2}+{\left(\frac{\partial v}{\partial t}\right)}^{2}+{\left(\frac{\partial w}{\partial t}\right)}^{2}]dxdydz$$

As mentioned earlier, the Winkler, Pasternak, and Kerr foundations are considered. The potential energy retained in the three elastic foundations $U_{f}$ can be denoted as [22]:

(11) $$\begin{array}{l} U_{f,\;Winkler}=\frac{1}{2}\int_{-b/2}^{b/2}\int_{-a/2}^{a/2}{\left.\left(K_{r}w^{2}\right)\right|}_{z=-h/2}dxdy\\ U_{f,\;Pasternak}=\frac{1}{2}\int_{-b/2}^{b/2}\int_{-a/2}^{a/2}{\left.\left\{K_{r}w^{2}+K_{g}\left[{\left(\frac{\partial w}{\partial x}\right)}^{2}+{\left(\frac{\partial w}{\partial y}\right)}^{2}\right]\right\}\right|}_{z=-h/2}dxdy\\ U_{f,\;Kerr}=\frac{1}{2}\int_{-b/2}^{b/2}\int_{-a/2}^{a/2}{\left.\left\{\frac{K_{u}\cdot K_{l}}{K_{u}+K_{l}}w^{2}+\frac{K_{s}\cdot K_{l}}{K_{u}+K_{l}}\left[{\left(\frac{\partial w}{\partial x}\right)}^{2}+{\left(\frac{\partial w}{\partial y}\right)}^{2}\right]\right\}\right|}_{z=-h/2}dxdy \end{array}$$

where $U_{f,\;Winkler}$, $\;U_{f,\;Pasternak}$ and $U_{f,\;Kerr}$ signify the potential energy retained from the three elastic foundations (Winkler, Pasternak, and Kerr foundations).

### 2.4. The Unified Solution with Admissible Displacement Functions

In this section, we conduct comparisons for seven different types of admissible functions. These derivation formulas of *i* (order) and ϕ (variable) can be denoted by $P_{i}(\phi )$. What needs to be considered is that the domains of ϕ in different polynomials are different. It can be found that:

(1) I-kind Chebyshev polynomials (Chebyshev I) [25]:

(12) $$P_{0}(\phi )=1,\;P_{1}(\phi )=\phi ,\;P_{i}(\phi )=2\phi P_{i-1}(\phi )-P_{i-2}(\phi ),\;i\geq 2,\;\phi \in [-1,1]$$

(2) II-kind Chebyshev polynomials (Chebyshev II) [25]:

(13) $$P_{0}(\phi )=1,\;P_{1}(\phi )=2\phi ,\;P_{i}(\phi )=2\phi P_{i-1}(\phi )-P_{i-2}(\phi ),\;i\geq 2,\;\phi \in [-1,1]$$

(3) Legendre polynomials [26]:

(14) $$P_{0}(\phi )=1,\;P_{1}(\phi )=2\phi ,\;P_{i}(\phi )=\frac{2i-1}{i}\phi P_{i-1}(\phi )-\frac{i-1}{i}P_{i-2}(\phi ),\;i\geq 2,\;\phi \in [-1,1]$$

(4) Orthogonal polynomials [27]:

(15) $$P_{0}(\phi )=1,\;P_{i}(\phi )=\frac{\psi_{i}(\phi )}{\sqrt{{\int_{0}^{1}\left[\psi_{i}(\phi )\right]}^{2}d\phi }},\;i\geq 1$$

where $\psi_{i}(\phi )$ are a combination of orthogonal polynomials. The recursive formula contained therein can be expressed as:

(16) $$\left\{\begin{array}{l} \psi_{1}(\phi )=1,\;\psi_{2}(\phi )=(\phi -B_{1})\psi_{1}(\phi )\\ \psi_{i+1}(\phi )=(\phi -B_{i})\psi_{i}(\phi )-C_{i}\psi_{i-1}(\phi ),\;i\geq 2 \end{array},\;\phi \in [0,1]\right.$$

where:

(17) $$B_{i}=\frac{\int_{0}^{1}\phi {\left[\psi_{i}(\phi )\right]}^{2}d\phi }{\int_{0}^{1}{\left[\psi_{i}(\phi )\right]}^{2}d\phi },\;C_{i}=\frac{\int_{0}^{1}\phi \psi_{i}(\phi )\psi_{i-1}(\phi )d\phi }{\int_{0}^{1}{\left[\psi_{i-1}(\phi )\right]}^{2}d\phi }$$

(5) I-kind modified Fourier series (modified Fourier I) [28]:

(18) $$P_{i}(\phi )=\left\{\begin{array}{l} \sin\frac{(i-3)\pi }{t}\phi ,\;1\leq i\leq 2\\ \cos\frac{(i-3)\pi }{t}\phi ,\;i>2 \end{array}\right.$$

In the *x* direction, t=a, ϕ∈[−a/2,a/2]; in the *y* direction, t=b, ϕ∈[−b/2,b/2]; in the *z* direction, t=h, ϕ∈[−h/2,h/2]. What is important to note is that the first two terms are complementary terms of two sinusoidal forms to ensure higher derivatives of the admissible displacement functions.

(6) II-kind modified Fourier series (modified Fourier II) [28]:

(19) $$P_{1}(\phi )=\phi {\left(\frac{\phi }{a}-1\right)}^{2},\;P_{2}(\phi )=\frac{\phi^{2}}{a}(\frac{\phi }{a}-1),\;P_{i}(\phi )=\cos\frac{(i-3)\pi }{a}\phi ,\;i\geq 3$$

In the *x* direction, t=a, ϕ∈[−a/2,a/2]; in the *y* direction, t=b, ϕ∈[−b/2,b/2]; in the *z* direction, t=h, ϕ∈[−h/2,h/2]. Similarly, to ensure the continuity of the function, two additional terms (*P*_1_ and *P*_2_) are adopted.

(7) Fourier–Bessel polynomials [29]:

(20) $$P_{0}=1,\;P_{1}=1+\phi ,\;P_{i}=\frac{2i}{\phi }P_{i-1}-P_{i-2},\;i\geq 2,\;\phi \in [0,1]$$

The displacement field of the FGP sandwich rectangular plate can be expressed as:

(21) $$\begin{array}{l} u(x,y,z)=\sum_{m=0}^{M}\sum_{n=0}^{N}\sum_{r=0}^{R}u_{mnr}T_{m}(\phi_{x})T_{n}(\phi_{y})T_{r}(\phi_{z})e^{i\omega t}\\ v(x,y,z)=\sum_{m=0}^{M}\sum_{n=0}^{N}\sum_{r=0}^{R}v_{mnr}T_{m}(\phi_{x})T_{n}(\phi_{y})T_{r}(\phi_{z})e^{i\omega t}\\ w(x,y,z)=\sum_{m=0}^{M}\sum_{n=0}^{N}\sum_{r=0}^{R}w_{mnr}T_{m}(\phi_{x})T_{n}(\phi_{y})T_{r}(\phi_{z})e^{i\omega t} \end{array}$$

where $u_{mnr},\;v_{mnr}$ and $w_{mnr}$ are the coefficients to be determined; *M*, *N* and *R* are the maximum values of *m*, *n* and *r*, respectively; ω is the angular frequency and *t* is the time; $T_{m}(\phi_{x}),\;T_{n}(\phi_{y})$ and $T_{r}(\phi_{z})$ are the polynomials of degree *m, n* and *r* in the *x, y* and *z* directions, respectively. Their expressions are:

(22) $$\begin{array}{l} T_{m}=[T_{0}(\phi_{x}),\;T_{1}(\phi_{x}),\;\cdots ,\;T_{\bar{m}}(\phi_{x}),\;\cdots ,\;T_{M}(\phi_{x})]\\ T_{n}=[T_{0}(\phi_{y}),\;T_{1}(\phi_{y}),\;\cdots ,\;T_{\bar{n}}(\phi_{y}),\;\cdots ,\;T_{N}(\phi_{y})]\\ T_{r}=[T_{0}(\phi_{z}),\;T_{1}(\phi_{z}),\;\cdots ,\;T_{\bar{r}}(\phi_{z}),\;\cdots ,\;T_{R}(\phi_{z})] \end{array}$$

The $\phi_{x}$,$\phi_{y}$ and $\phi_{z}$ are the coordinate points transformed as described above in the *x, y* and *z* directions, respectively. They are obtained from linear transformations of *x, y* and *z*, since different polynomials are defined in diverse intervals.

For Chebyshev I, II and Legendre polynomials:

(23) $$\phi_{x}=2x/a-1,\;\phi_{y}=2y/b-1,\;\phi_{z}=2z/h-1$$

For orthogonal polynomials as well as the Fourier–Bessel series:

(24) $$\phi_{x}=2x/a,\;\phi_{y}=2y/b,\;\phi_{z}=2z/h$$

For modified Fourier I and modified Fourier II:

(25) $$\phi_{x}=x,\;\phi_{y}=y,\;\phi_{z}=z$$

After that, the admissible displacement functions can be expressed in a unified form as:

(26) $$u=U\cdot G_{u},\;v=V\cdot G_{v},\;w=W\cdot G_{w}$$

where

(27) $$U=\left\{T_{0}(\phi_{x})T_{0}(\phi_{y})T_{0}(\phi_{z}),\;\cdots ,\;T_{m}(\phi_{x})T_{n}(\phi_{y})T_{r}(\phi_{z}),\;\;\cdots ,T_{M}(\phi_{x})T_{N}(\phi_{y})T_{R}(\phi_{z})\right\}$$

(28) U=W=V

(29) $$\begin{array}{l} G_{u}=\left\{u_{000},\;\cdots ,\;u_{00R},\;\cdots ,u_{0NR},\;\cdots ,u_{MNR}\right\}e^{i\omega t}\\ G_{v}=\left\{v_{000},\;\cdots ,\;v_{00R},\;\cdots ,v_{0NR},\;\cdots ,v_{MNR}\right\}e^{i\omega t}\\ G_{w}=\left\{w_{000},\;\cdots ,\;w_{00R},\;\cdots ,w_{0NR},\;\cdots ,w_{MNR}\right\}e^{i\omega t} \end{array}$$

Based on the previous text, the Rayleigh–Ritz method can be used for the solution procedure. Then, another form of the Lagrangian energy function of the FGP sandwich rectangular plate can be written as:

(30) $$L=T-U_{s}-U_{bc}-U_{f}$$

After that, the partial differential of *L* with respect to the coefficients $\vartheta (=u_{mnr},\;v_{mnr}\;\mathrm{and}\;w_{mnr})$ to be determined is zero, as below:

(31) $$\frac{\partial L}{\partial \vartheta }=0,\;\;\vartheta =u_{mnr},\;v_{mnr},\;w_{mnr}$$

By integrating Equations (21) and (30) into Equation (31), the motion equation of the FGP sandwich rectangular plates can be expressed as:

(32) $$(K-\omega^{2}M)G=0$$

where *K* represents the stiffness matrix in regard to potential energy and strain energy, retained from boundaries and elastic foundations. *M* can be signified as the mass matrix relevant to the kinetic energy; $G={\left[G_{u},\;G_{v},\;G_{w}\right]}^{T}$.

More details on *K* or *M* can be found in Appendix A.

## 3. Numerical Results

In this section, the numerical simulation results of the FGP sandwich rectangular plates resting on Winkler, Pasternak, and Kerr foundations are presented. As stated, in all situations, the material properties of the metal are as follows: *E_m_* = 70 Gpa; *ρ_m_* = 2707 kg/m^3^; *ν_m_* = 0.3, where the aluminum (Al) is adopted [36]. In addition, the ceramic part of the functionally gradient porous sandwich rectangular plate is alumina (Al_2_O_3_) [36], and its material properties are *E_c_* = 380 Gpa; *ρ_c_* = 3800 kg/m^3^; *ν_c_* = 0.3. The thickness ratio of the plates can be expressed as *h*_1_–*h*_2_–*h*_3_, while *h_i_* (*i* = 1, 2 and 3) represents the proportion of the rectangular plate rather than the specific length. In this study, the dimensionless frequency parameter Ω is defined as $\Omega =\omega a^{2}/h\sqrt{\rho_{m}/E_{m}}$, where *a* represents the length of the rectangular plate. Then, the parameters studied contain the porosity coefficient *e*_0_, geometric parameters (*a*/*b*, *h*/*b* and thickness ratio), porosity distribution *P_i_* of the rectangular plate, weight fraction *k*, and parameters concerning the elastic foundations.

At the same time, we also consider arbitrary boundary conditions. By properly adjusting the boundary spring stiffness values *k_i_* (*i = u*, *v* and *w*), several boundary conditions, including free (*F*), simply supported (*S*), elastic (*E*), and clamped (*C*), can be conveniently achieved, whose values are presented below:

(33) $$\begin{array}{l} F:k_{u}=k_{v}=k_{w}=0;\\ S:k_{u}=0,\;k_{v}=k_{w}=10^{18};\\ C:k_{u}=k_{v}=k_{w}=10^{18}; \end{array}$$

where the values of the boundary springs and elastic foundation will be explained later. A four-string letter is used to describe the boundary conditions at each edge of the rectangular plate. For example, *CFSE* represents the *C*, *F*, *S*, and *E* boundary conditions at edges *x* = 0, *y* = 0, *x* = *a* and *y* = *b*, respectively. With respect to the elastic foundations, to simplify the model and formula, the dimensionless parameters can be written as [22]:

(34) $$\begin{array}{l} {\bar{K}}_{l}=K_{l}D/b^{4}\\ {\bar{K}}_{u}=K_{u}D/b^{2}\\ {\bar{K}}_{s}=K_{s}D/b^{4} \end{array}$$

where *b* represents the width of the rectangular plate, and *D* indicates the flexural stiffness, which can be expressed as $D=(E_{c}h^{3})/12(1-\nu^{2})$.

### 3.1. Comparison, Validation, and Convergence Studies

**Figure 2:**
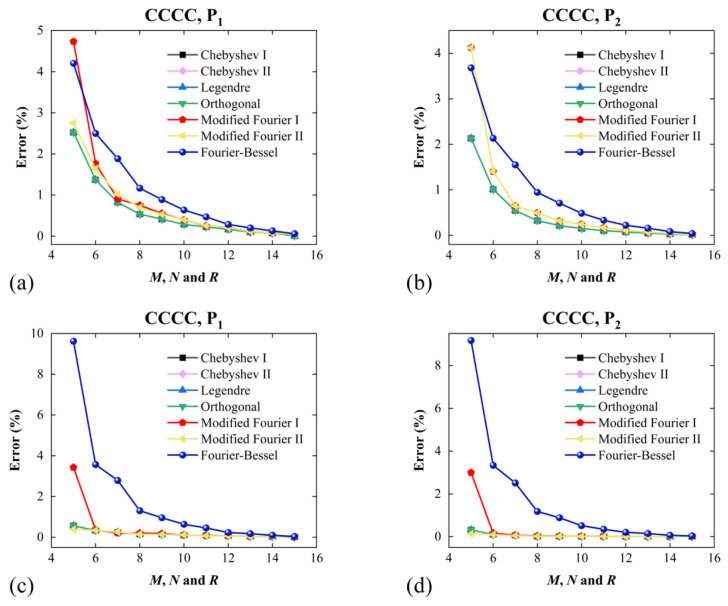
Variations in *Error* with truncated numbers (*M*, *N* and *R*) for FGP sandwich plates, where seven admissible displacement functions are employed. Boundary conditions and porosity distribution: (**a**) *CCCC* and *P*_1_, (**b**) *SSSS* and *P*_1_, (**c**) *CCCC* and *P*_2_ and (**d**) *SSSS* and *P*_2_.

Different admissible functions used to discretize the actual displacement of FGP sandwich plates may have different effects on consistency and computational efficiency. The comparison and selection of the functions can make the solving procedure for the free vibration of FGP sandwich plates more accurate and efficient. In this part, different admissible functions are adopted in the algorithm to investigate the performance in this study. Figure 2 shows the convergence characteristics of the frequency of the functionally gradient porous sandwich rectangular plates regarding the truncated numbers (*M* = *N* = *R*) with different admissible functions under *CCCC* and *SSSS* boundary conditions. Selecting the fundamental frequency (first mode) Ω of the functionally gradient porous sandwich rectangular plates as the object, the value of *Error* is expressed as $Error=\left|\Omega -\Omega_{M=N=R=16}\right|/\Omega_{M=N=R=16}$. The material properties and geometric parameters are *a*/*b* = 1; *h*/*b* = 0.3; *e*_0_ = 0.2; porosity distribution: *P*_1_; thickness ratio: 1-1-1.

In general, the following aspects can be summarized: Firstly, although the boundary conditions are different, cases of the Fourier–Bessel polynomial have the largest number of terms when it reaches convergence; next are modified Fourier I and II. For the other cases (Chebyshev I and II, Legendre, and orthogonal polynomials), they show similar convergence characteristics, which have fast convergence properties. For *M* = *N* = *R* = 13, the errors of all are no more than 0.01%.

**Figure 3:**
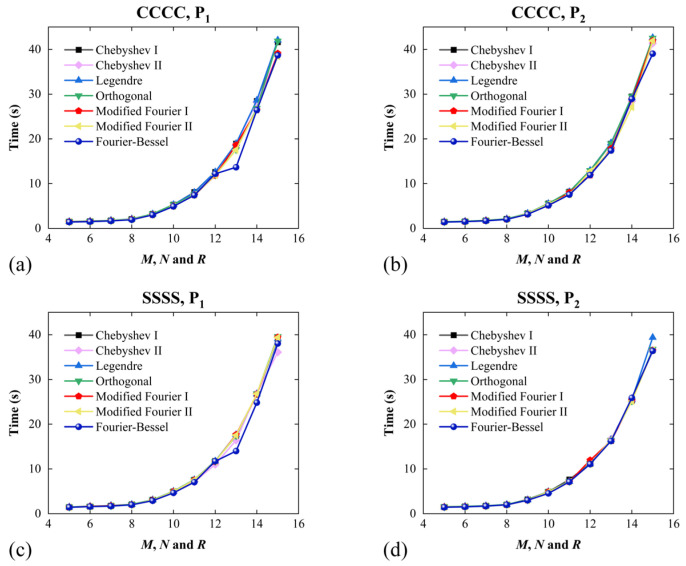
Variations in time with truncated numbers (*M, N* and *R*) for FGP sandwich plates, where seven admissible displacement functions are employed. Boundary conditions and porosity distribution: (**a**) *CCCC* and *P*_1_, (**b**) *SSSS* and *P*_1_, (**c**) *CCCC* and *P*_2_ and (**d**) *SSSS* and *P*_2_.

Through the relations between computational time and truncated numbers, a comparison of the computational time of the seven different types of admissible displacement functions was conducted, as shown in Figure 3. In addition, the relevant parameters are consistent with those in Figure 2. It can be observed that the computational time of modified Fourier I and II is the longest, while that of the other admissible displacement functions is similar at the same value.

According to the simulation results, the following conclusions may be drawn. Compared with the last three cases (Fourier–Bessel, Modified Fourier I and II polynomials), the time and truncated number required to reach convergence in the first four cases (Chebyshev I and II, Legendre and orthogonal polynomials) are smaller, and they have similar convergence characteristics. So, it can be considered that the calculation efficiency of the first four polynomials is higher. Based on this, in the subsequent calculations, the Chebyshev I polynomial is selected as the admissible displacement function of FGP sandwich rectangular plates. Moreover, the number of truncated numbers is selected as *M* = *N* = *R* = 13, where their error is less than 0.01% in Figure 2.

For the purpose of verifying the precision of the algorithm, Table 1 gives a comparison of the first mode of the functionally gradient porous sandwich rectangular plate calculated by the presented method based on the results of [37,38]. The method from [37] used the Chebyshev polynomial to multiply by appropriate functions to expand the displacement of the rectangular plate to achieve the basic boundary conditions, while [38] obtained the partial differential equation of the motion control equation using third-order shear deformation plate theory and the Hamilton principle. The geometric and material properties are *a*/*b* = 1; *h*/*b* = 0.1; *e*_0_ = 0; six thickness ratios (1-0-1, 2-1-2, 2-1-1, 1-1-1 and 2-2-1) are taken into consideration. The results prove the accuracy of the unified solution proposed earlier through the results of [37,38]. Moreover, in order to further verify the accuracy of the unified solution, the first seven natural frequencies of the rectangular plate are studied. Table 2 shows the calculation results of the first seven natural frequencies under different boundary conditions (*SSSS*, *CCCC* and *FFFF*) and with different properties [39]. By comparing the results, the maximum error value (absolute value) is 0.366%, which proves the effectiveness of the algorithm for free vibration analysis of the plates. Based on previous comparisons, Table 3 shows the first four modes of the FGP sandwich plate calculated in this study and obtained via the finite element method (FEM), which can further verify the algorithm.

**Figure 4:**
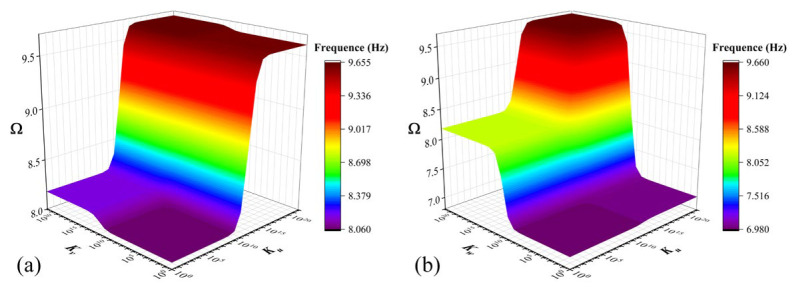
Dependence of Ω on boundary spring parameters: (**a**) *k_u_* and *k_v_*; (**b**) *k_u_* and *k_w_*.

Since the method has been verified, before analyzing the vibration characteristics of FGP sandwich rectangular plates, the convergence characteristics of boundary spring stiffness should be studied. The variations in the fundamental frequency Ω of the FGP sandwich rectangular plates with the changing stiffness of the boundary springs *k_u_* and *k_v_* (or *k_w_*) are provided in Figure 4. The material properties and geometric parameters of FGP sandwich rectangular plates involved are shown as follows: *a*/*b* = 1; *h*/*b* = 0.3; *e*_0_ = 0.2; porosity distribution: *P*_1_; thickness ratio: 1-1-1. For any case, when the stiffness of the two boundary springs changes, the remaining spring stiffness value is set as 10^23^. It can be seen that Ω changes dramatically when the boundary spring stiffness value *k_i_* (*i* = *u*, *v* and *w*) changes between 10^10^ and 10^15^. Based on this, the mutation region can be regarded as the interval of boundary spring stiffness values corresponding to elastic restraint. Therefore, by selecting the boundary spring stiffness value within this interval, the simulation of elastic boundary conditions can be obtained. In addition, when both *k_u_* and *k_v_* (or *k_w_*) are not less than 10^15^, a convergence interval of the maximum frequency can be observed. In summary, the boundary spring stiffness corresponding to elastic boundary conditions (*E*) is *k_u_* = *k_v_* = *k_w_* = 10^12^; that corresponding to the clamped boundary condition (*C*) is *k_u_* = *k_v_* = *k_w_* = 10^18^.

### 3.2. Benchmark Results

**Figure 5:**
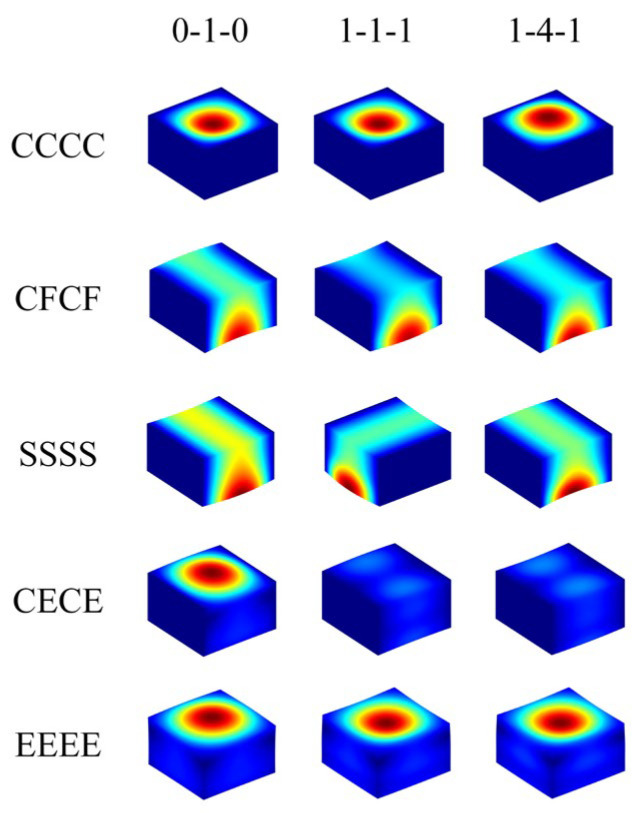
Several mode shapes for FGP sandwich rectangle plates resting on Pasternak foundation with different boundary conditions and thickness ratios.

**Figure 6:**
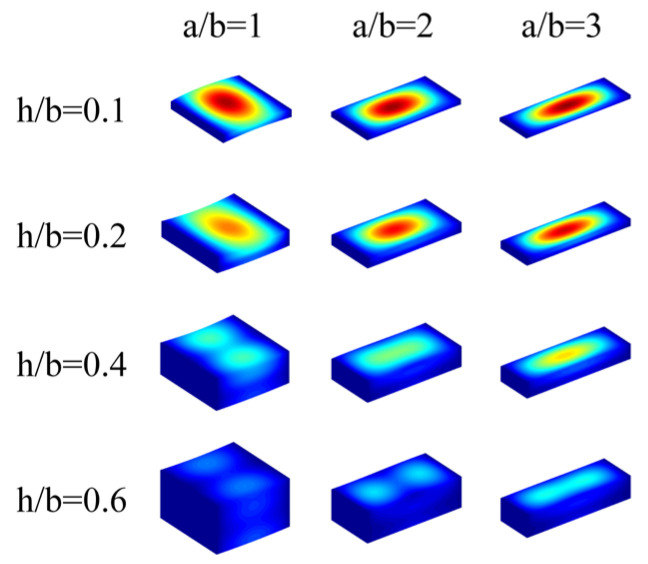
Several mode shapes for FGP sandwich rectangle plates resting on Pasternak foundations with different *a*/*b* and *h*/*b*.

In this section, some other results of the free vibration of FGP sandwich rectangular plates resting on an elastic foundation are reported. Table 4 shows the fundamental frequency Ω of FGP sandwich rectangular plates with different porosity distributions, geometric parameters (*h*/*b*), thickness ratios, and boundary conditions resting on the Pasternak foundation. The parameters of the cases mentioned are as follows: *a*/*b* = 1; *e*_0_ = 0.2; *k_r_* = *k_g_* = 10^12^. Five types of boundary conditions are selected (*CCCC*; *CFCF*; *SSSS*; *CECE*; *EEEE*). The effect of porosity distributions and thickness ratios on the fundamental frequency of FGP sandwich rectangular plates is not significant. Under the same boundary condition, geometric parameter, porosity distribution, and thickness ratio, the case with a thickness ratio of 0-1-0 consistently corresponds to the largest fundamental frequency. Furthermore, the effects of porosity distributions are much more complex, and for different geometric parameters and boundary constraints, the highest fundamental frequency occurs at different porosity distributions. Like the case of *a*/*b* = 1, the largest fundamental frequency occurs with porosity distribution *P*_3_ under the *CCCC* boundary condition, while under the *EEEE* boundary condition, the largest value comes with *P*_1_. The increasing aspect ratios of FGP sandwich rectangular plates lead to an increase in the fundamental frequency, but it does not affect their variation patterns under different conditions. Figure 5 provides various mode shapes for FGP sandwich rectangular plates with porosity distribution *P*_1_ and different thickness ratios under different boundary constraints, while the mode shapes with porosity distribution *P*_1_ and different geometric parameters resting on the Pasternak foundation are shown in Figure 6.

Table 5 presents the fundamental frequency of FGP sandwich rectangular plates with different volume fraction indices *k*, porosity coefficients *e*_0_, and boundary conditions resting on the Pasternak foundation. The relevant parameters are as follows: *a*/*b* = 1; *h*/*b* = 0.4; porosity distribution: *P*_1_; thickness ratio: 1-1-1; and elastic foundation parameters: *K_r_* = *K_g_* = 10^15^. Obviously, regardless of different boundary conditions, for sandwich rectangular plates with a functionally graded core, the fundamental frequency decreases with *k* and *e*_0_ increasing.

In the following part, the frequencies of FGP sandwich rectangular plates resting on Winkler/Pasternak with different combinations of *K_r_* and *K_g_*, along with the Kerr foundation with different *K_l_*, *K_s_* and *K_u_*, are presented in Table 6 and Table 7, respectively. The analysis is conducted depending on the first four frequencies, and the parameters in Table 6 and Table 7 are consistent with those in Table 5. For the Winkler/Pasternak foundation, in all cases, the largest Ω occurs at (*K_r_*, *K_g_*) = (10^10^, 10^10^), followed by (*K_r_*, *K_g_*) = (0, 10^10^) and then (*K_r_*, *K_g_*) = (10^10^, 0), despite the presence of four frequencies and different boundary conditions (Table 5).

Regarding the Kerr foundation, as (*K_u_*, *K_s_*) changes in a sequence of (10^8^, 0), (10^8^, 10^8^) and (10^11^, 10^8^), the Ω also increases (Table 7). In addition, for the same values of *K_u_* and *K_s_*, the value of Ω (of any frequency) at *K_l_* = 10^11^ is no larger than that at *K_l_* = 10^8^ under any boundary condition.

### 3.3. Parametric Study

In this part, parametric analysis is conducted on the vibration characteristics of FGP sandwich rectangular plates based on an elastic foundation, geometric parameters (aspect ratio and thickness ratio), and material properties (volume fraction index *k* and porosity coefficient *e*_0_), respectively.

**Figure 7:**
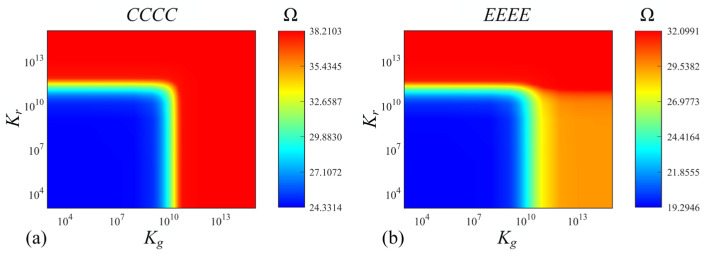
Contour plots of Ω depending on *K_r_* and *K_g_* for FGP sandwich rectangle plates resting on Pasternak foundation: (**a**) *CCCC* and (**b**) *EEEE* boundary condition.

**Figure 8:**
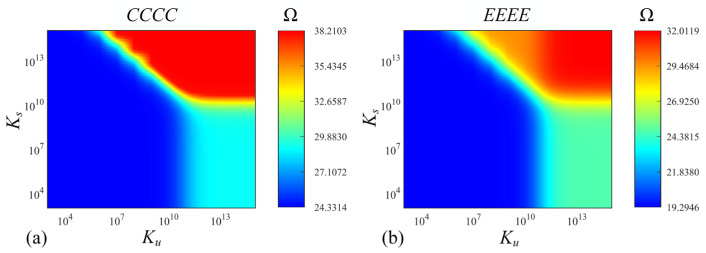
Contour plots of Ω depending on *K_u_* and *K_s_* for FGP sandwich rectangle plates resting on Kerr foundation: (**a**) *CCCC* and (**b**) *EEEE* boundary condition.

Figure 7 and Figure 8 illustrate the effects of parameters of the Pasternak foundation (*K_r_* and *K_g_*) and Kerr foundation (*K_u_*, *K_s_* and *K_l_*) on the fundamental frequency Ω of functionally graded porous sandwich rectangular plates, where both the *CCCC* and *EEEE* boundary constraints are taken into consideration. The geometric parameters and material properties are as follows: *a*/*b* = 2; *h*/*b* = 0.4; *e*_0_ = 0.4; *k* = 1; porosity distribution: *P*_1_; thickness ratio: 1-1-1. Under the *CCCC* boundary condition resting on the Pasternak foundation (Figure 7a), when *K_g_* is less than 2.5 × 10^9^ and *K_r_* is less than 4 × 10^10^, the Ω remains constant (minimum value). Meanwhile, when within a range of 2.5 × 10^9^ < *K_g_* < 4 × 10^10^ and *K_r_* < 4 × 10^11^ (or *K_g_* < 4 × 10^10^ and 4 × 10^10^ < *K_g_* < 4 × 10^11^), the Ω rapidly aggrandizes. As *K_g_* and *K_r_* continue to increase, the tends to reach a constant maximum value. When it comes to the *EEEE* boundary condition (Figure 7b), the major trend of Ω is mostly consistent with that under the *CCCC* boundary constraint, while the difference is that when *K_g_* is greater than 4 × 10^11^, the convergence region of the maximum value reached by Ω is smaller (compared to *CCCC* boundary constraints). Under the *CCCC* boundary condition (Figure 8a) and *SSSS* boundary condition (Figure 8b) resting on the Kerr foundation, the effects of *K_u_* and *K_s_* on FGP sandwich rectangular plates are more complex than that resting on Pasternak foundations. Similarly, there are three convergence regions (minimum, relative maximum, and maximum values) and one irregular transition region (within a range of 4 × 10^6^ < *K_u_* < 4 × 10^11^ and *K_s_* > 1 × 10^10^) of Ω in the contour plots for both boundary conditions resting on the Kerr foundation. The differences are that the effects of *K_u_* and *K_s_* in the Kerr foundation on Ω are more sensitive in this transition region, which is affected by the syntactic effect of *K_u_* and *K_s_*.

**Figure 9:**
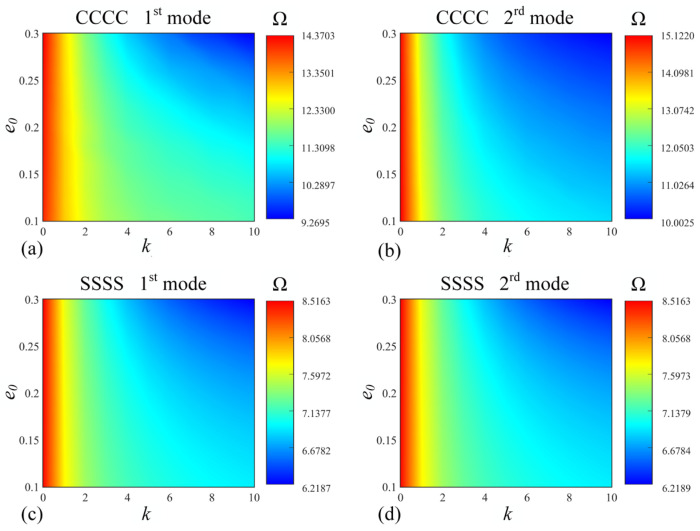
Variations in (**a**,**b**) first mode Ω and (**c**,**d**) second mode Ω with *k* and *e*_0_ for FGP sandwich rectangle plates resting on Pasternak foundation with different boundary conditions: (**a**,**c**) *CCCC* and (**b**,**d**) *EEEE* boundary conditions.

In order to investigate the effects of the porosity coefficient *e*_0_ and volume fraction index *k* on the vibration characteristics of FGP sandwich rectangular plates, Figure 9 shows the values of the first and second frequencies under different boundary conditions (*CCCC* and *SSSS*) resting on the Pasternak foundation as *e*_0_ and *k* vary. The relevant parameters are *a*/*b* = 2; *h*/*b* = 0.4; *e*_0_ = 0.4; *k* = 1; porosity distribution: *P*_1_; thickness ratio: 1-1-1. For accuracy, the parameters of the elastic foundation are taken as *K_r_* = *K_g_* = 10^15^. In addition, the values of 0.1 ≤ *e*_0_ ≤ 0.3 and 0 ≤ *k* ≤ 10 are selected in this study. Firstly, it can be clearly observed that the Ω remains constant when *k* = 0, which is due to the fact that the core of the rectangular plate is not a functionally graded material. Then, the Ω decreases as *e*_0_ and *k* increase, while it is less noticeable when *e*_0_ and *k* are small, as it does not reach the corresponding thresholds. Overall, for different boundary conditions, the trends of the effects of *e*_0_ and *k* are generally similar.

**Figure 10:**
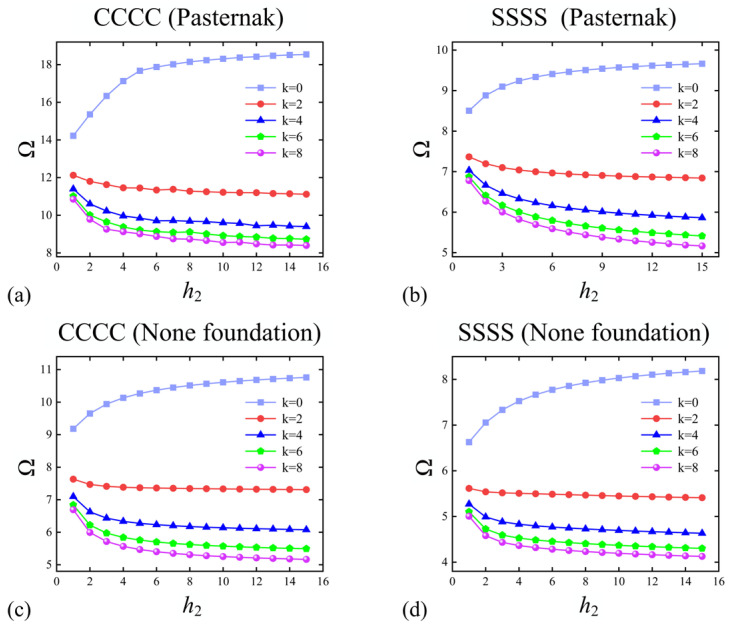
Variations in Ω with *h*_2_ for 1-*h*_2_-1 FGP sandwich rectangle plates resting on (**a**,**b**) Pasternak and (**c**,**d**) non-elastic foundation: (**a**,**c**) *CCCC* and (**b**,**d**) *SSSS* boundary condition.

The variation in the fundamental frequency Ω of FGP sandwich rectangular plates with core thickness *h*_2_ and volume fraction index *k* under the *CCCC* boundary condition and *SSSS* boundary condition resting on the non-elastic and Pasternak foundations can be observed in Figure 10. The parameters not studied are consistent with those in Figure 9, so the thickness ratio of the sandwich plate can be expressed as 1-*h*_2_-1. From Figure 10, it can be observed that when *k* = 0, indicating that the plate is not made of functionally graded material and the core layer is just a porous alumina layer, the Ω aggrandizes as *h*_2_ increases. When *k* > 0, the Ω decreases with the increase in *h*_2_ and *k*. The trends of the fundamental frequency of FGP sandwich rectangular plates with respect to *h*_2_ and *k* are generally consistent under different boundary conditions and resting on non-elastic or elastic foundations, which is not surprising.

**Figure 11:**
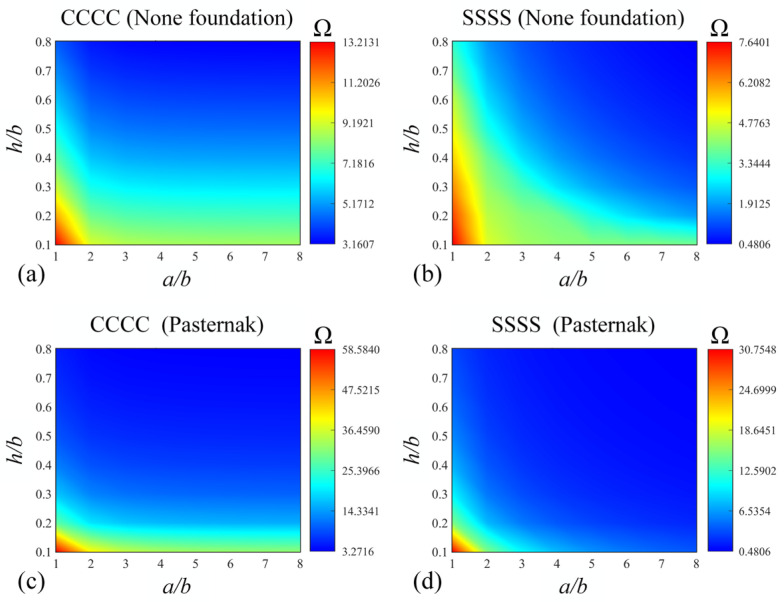
Variations in Ω with *a*/*b* and *h*/*b* for FGP sandwich rectangle plates resting on (**a**,**b**) non-elastic and (**c**,**d**) Pasternak foundations under different boundary conditions: (**a**,**c**) *CCCC* and (**b**,**d**) *SSSS* boundary conditions.

Figure 11 shows the effects of geometric parameters (*a*/*b* and *h*/*b*) on the fundamental frequency Ω of FGP sandwich rectangular plates under the *CCCC* boundary condition and *SSSS* boundary condition, resting on the non-elastic and Pasternak foundations. The relevant parameters are *e*_0_ = 0.2; *k* = 2; porosity distribution: *P*_1_; thickness ratio: 1-1-1; elastic foundation parameters: *K_r_ = K_g_* = 10^15^. As the changes in *b* will affect the normalization of elastic foundation parameters *K_r_* and *K_g_*, *a*/*b* and *h*/*b* are chosen as the variables of interest, with *b* held constant. Regardless of whether the FGP sandwich plate is resting on an elastic foundation or not, for different boundary conditions, the value of Ω decreases as *a*/*b* and *h*/*b* increase; moreover, there are two convergence regions (minimum and maximum values). It is also demonstrated that the maximum value of Ω occurs at the smallest values of *a*/*b* and *h*/*b*, while the minimum value of Ω occurs at the largest values of *a*/*b* and *h*/*b*. It can be found that the effects of *a*/*b* and *h*/*b* on the fundamental frequency of FGP sandwich plates are similar. The dissemination of vibration waves in rectangular plates with different geometries (*a*/*b* and *h*/*b*) is different, which makes vibration frequencies different. When *a*/*b* is close to 1, the interaction is the strongest, causing that the vibration frequency to be the highest. When it comes to *h*/*b*, due to the increase in thickness, the relative weight of FGP sandwich rectangular plates increases, so the frequency decreases. Furthermore, due to the buffering of the elastic foundation, the effect of *a*/*b* and *h*/*b* on the vibration frequencies of rectangular plates is greatly reduced.

## 4. Conclusions

A unified method for the free vibration analysis of FGP sandwich rectangular plates under arbitrary boundary conditions resting on various elastic foundations is presented. The innovative points of this study were highlighted, and the following conclusions can be drawn:

(1) The unified method for FGP sandwich rectangular plates based on elastic foundations based on three-dimensional elastic theory was proposed in this study. Because the algorithm does not require preset conditions for calculation, it is widely used and not limited to a specific structure. By comparing with FEM and references, it can be found that the unified method has good accuracy and convergence effects.

(2) Seven admissible displacement functions were considered in the unified solution based on the Rayleigh–Ritz procedure. The results indicate that Chebyshev I and II, Legendre, and orthogonal polynomials exhibit similar convergence performances, with little difference in computation efficiency among these polynomials.

(3) Winkler, Pasternak, and Kerr foundations are considered in the study of the FGP sandwich rectangular plates, and the effects of parameters of elastic foundations on vibration characteristics are considered.

(4) Different combinations of porosity distributions, porosity coefficients, volume fraction indices, thickness ratios, and geometric parameters are considered, and the effects on the vibration characteristics (fundamental frequency Ω) of FGP sandwich rectangular plates are well demonstrated. The impact of porosity distributions on vibration frequency is complex and depends on different boundary conditions. Among the parameters, the porosity coefficient has a relatively small effect (vibration frequency exhibits small variations). In general, the effects of the parameters studied on FGP sandwich rectangular plates tend to reach a convergence region with variables, unaffected by various boundary conditions. With an increase in the length-to-width ratio and thickness-to-width ratio, the vibration frequency of the rectangular plates will decrease. When the core layer is the gradient layer, the vibration frequency of rectangular plates decreases with the increasing thickness of the core layer, while the core layer consists of uniform material and will increase with increasing thickness. In addition, an increase in the volume fraction index will reduce the vibration frequency of rectangular plates.

## Figures and Tables

**Table 1:** Comparison of Ω (Hz) (fundamental frequency) for simply supported FGM sandwich plates with homogeneous core and FGM face sheet with different thickness ratios and *k*.

*k*	Methods	Thickness Ratio					
		1-0-1	2-1-2	2-1-1	1-1-1	2-2-1	1-2-1
0	Ref. [37]	1.824	1.824	1.824	1.824	1.824	1.824
	Ref. [38]	1.827	1.827	--	1.827	1.827	1.827
	Present	1.827	1.827	1.827	1.827	1.827	1.827
0.5	Ref. [37]	1.444	1.484	1.506	1.519	1.547	1.575
	Ref. [38]	1.446	1.486	--	1.521	1.549	1.577
	Present	1.446	1.486	1.508	1.521	1.549	1.577
1	Ref. [37]	1.243	1.300	1.333	1.353	1.396	1.435
	Ref. [38]	1.245	1.302	--	1.355	1.398	1.441
	Present	1.245	1.302	1.335	1.355	1.398	1.441
5	Ref. [37]	0.946	0.982	1.030	1.045	1.109	1.174
	Ref. [38]	0.945	0.981	--	1.045	1.110	1.176
	Present	0.944	0.981	1.029	1.045	1.109	1.175
10	Ref. [37]	0.928	0.943	0.992	0.996	1.061	1.123
	Ref. [38]	0.927	0.941	--	0.995	1.061	1.125
	Present	0.926	0.940	0.989	0.995	1.060	1.124

**Table 2:** Comparison of Ω (Hz) for FGP square plates with various porosity distributions under different boundary conditions.

Boundary Conditions	*h*/*a*	Methods	Mode						
			1	2	3	4	5	6	7
*SSSS*	0.1	Present	19.090	45.619	45.619	64.383	64.383	70.104	85.503
		Ref. [39]	19.098	45.636	45.636	64.384	64.384	70.149	85.500
	0.2	Present	17.526	32.192	32.192	38.483	38.483	45.526	55.787
		Ref. [39]	17.528	32.192	32.192	38.488	38.488	45.526	55.802
	0.5	Present	12.426	12.877	12.877	18.210	23.008	23.008	25.753
		Ref. [39]	12.426	12.877	12.877	18.210	23.009	23.009	25.753
*CCCC*	0.1	Present	32.904	62.866	62.866	88.179	103.951	104.945	123.719
		Ref. [39]	33.009	63.043	63.043	88.411	104.280	105.290	123.730
	0.2	Present	26.966	47.192	47.192	61.961	61.961	63.449	72.407
		Ref. [39]	27.065	47.346	47.346	62.000	62.000	63.635	72.604
	0.5	Present	15.305	24.086	24.086	24.832	24.832	29.377	31.519
		Ref. [39]	15.358	24.136	24.136	24.866	24.866	29.379	31.578
*FFFF*	0.1	Present	12.738	18.955	23.346	31.975	31.975	55.498	55.498
		Ref. [39]	12.728	18.956	23.346	31.965	31.965	55.493	55.493
	0.2	Present	11.711	17.433	21.252	27.649	27.649	40.192	42.775
		Ref. [39]	11.710	17.433	21.252	27.648	27.648	40.192	42.775
	0.5	Present	8.780	12.515	14.961	16.072	17.030	17.030	17.631
		Ref. [39]	8.7801	12.515	14.962	16.072	17.030	17.030	17.632

**Table 3:** Comparison of first four modes with FEM under different boundary conditions.

Boundary Conditions	Methods	Mode			
		1	2	3	4
*FFFF*	FEM	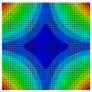	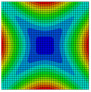	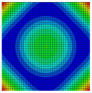	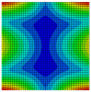
	Present	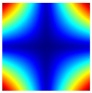	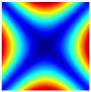	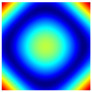	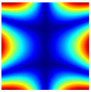
*SSSS*	FEM	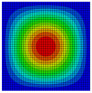	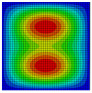	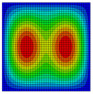	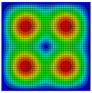
	Present	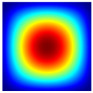	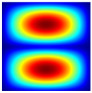	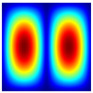	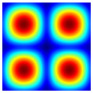
*CCCC*	FEM	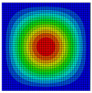	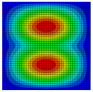	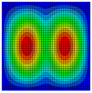	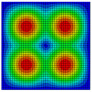
	Present	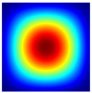	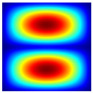	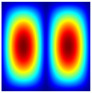	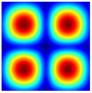

**Table 4:** Non-dimensional fundament frequency Ω for FGP sandwich rectangular plates with various aspect ratios (*h*/*b*) and porosity distributions resting under different boundary conditions on Pasternak foundation.

Type *a*/*b* = 1	*h*/*b*	Boundary Condition														
		CCCC			CFCF			SSSS			CECE			EEEE		
		0-1-0	1-1-1	1-4-1	0-1-0	1-1-1	1-4-1	0-1-0	1-1-1	1-4-1	0-1-0	1-1-1	1-4-1	0-1-0	1-1-1	1-4-1
*P*_1_	0.1	60.94	60.63	60.76	29.06	29.03	29.04	31.84	31.80	31.82	48.81	47.20	47.94	45.96	43.89	44.85
	0.2	29.98	29.28	29.58	14.51	14.45	14.48	15.88	15.81	15.84	24.11	23.10	23.54	22.68	21.46	22.00
	0.4	13.59	12.83	13.30	7.18	7.03	7.09	7.86	7.71	7.77	11.77	11.00	11.32	11.12	10.36	10.67
	0.6	8.13	7.90	8.07	4.65	4.40	4.50	5.14	4.90	5.00	7.60	7.02	7.27	7.04	6.69	6.93
*P*_2_	0.1	61.02	60.71	60.86	29.07	29.04	29.05	31.84	31.81	31.83	48.14	47.04	47.56	45.08	43.66	44.34
	0.2	30.16	29.45	29.81	14.53	14.47	14.50	15.89	15.83	15.86	23.86	23.08	23.45	22.33	21.39	21.84
	0.4	14.39	12.98	13.80	7.21	7.07	7.14	7.88	7.75	7.82	11.75	11.08	11.39	11.03	10.38	10.68
	0.6	8.66	7.99	8.29	4.72	4.46	4.59	5.18	4.96	5.08	7.73	7.11	7.42	7.32	6.75	7.00
*P*_3_	0.1	61.02	60.71	60.87	29.07	29.04	29.05	31.84	31.81	31.83	48.11	47.03	47.54	45.04	43.64	44.31
	0.2	30.16	29.46	29.82	14.53	14.47	14.50	15.89	15.83	15.86	23.85	23.07	23.44	22.31	21.39	21.83
	0.4	14.42	12.99	13.76	7.21	7.07	7.15	7.88	7.75	7.82	11.74	11.08	11.40	11.02	10.37	10.68
	0.6	8.69	7.95	8.25	4.72	4.46	4.59	5.18	4.97	5.08	7.72	7.12	7.41	7.32	6.75	7.01

**Table 5:** Dimensionless frequency Ω (first mode) for FGP sandwich rectangle plates with various volume fraction indices *k* and porosity coefficients *e*_0_ under different boundary conditions resting on Pasternak foundation.

*k*	*e*_0_	Boundary Condition				
		CCCC	CSCS	CECE	EEEE	CFCF
0	0	13.973	8.480	11.894	10.889	7.757
	0.05	14.034	8.486	11.926	10.934	7.763
	0.1	14.096	8.492	11.957	10.979	7.769
	0.3	14.343	8.517	12.080	11.159	7.792
1	0	12.931	7.771	11.092	10.348	7.097
	0.05	12.925	7.759	11.083	10.357	7.086
	0.1	12.911	7.745	11.070	10.362	7.073
	0.3	12.751	7.665	10.957	10.330	7.000
2	0	12.474	7.490	10.736	10.079	6.837
	0.05	12.414	7.466	10.700	10.063	6.814
	0.1	12.337	7.437	10.655	10.039	6.788
	0.3	11.752	7.250	10.332	9.823	6.617
5	0	12.011	7.188	10.355	9.781	6.558
	0.05	11.874	7.145	10.282	9.732	6.519
	0.1	11.700	7.093	10.192	9.667	6.471
	0.3	10.255	6.632	9.488	9.116	6.059
10	0	11.819	7.047	10.186	9.648	6.430
	0.05	11.642	6.994	10.095	9.582	6.382
	0.1	11.412	6.929	9.981	9.496	6.322
	0.3	9.325	6.219	8.820	8.295	5.694

**Table 6:** Dimensionless frequency Ω (first mode) for FGP sandwich rectangle plates resting on Pasternak foundation with different combinations of *K_r_* and *K_g_*.

*K* *_r_*	*K_g_*	Mode Number	Boundary Condition				
			CCCC	CECE	CSCS	EEEE	CFCF
0	0	1	8.176	7.378	7.034	6.488	5.551
		2	12.813	10.840	7.711	10.206	5.782
		3	12.813	12.211	12.233	10.206	7.383
		4	14.131	12.385	12.417	11.594	8.896
0	10^10^	1	8.198	7.400	7.056	6.510	5.568
		2	12.825	10.846	7.711	10.211	5.809
		3	12.825	12.239	12.262	10.211	7.388
		4	14.164	12.397	12.422	11.621	8.930
10^10^	0	1	8.177	7.379	7.036	6.490	5.552
		2	12.814	10.840	7.711	10.206	5.783
		3	12.814	12.212	12.233	10.206	7.383
		4	14.132	12.386	12.417	11.594	8.896
10^10^	10^10^	1	8.199	7.401	7.057	6.512	5.570
		2	12.825	10.846	7.711	10.211	5.811
		3	12.825	12.240	12.262	10.211	7.388
		4	14.164	12.397	12.422	11.622	8.930

**Table 7:** Dimensionless frequency Ω (first mode) for FGP sandwich rectangle plates resting on Kerr foundation with different combinations of *K_l_*, *K_u_* and *K_s_*.

*K_l_*	*K_u_*	*K_s_*	Mode Number	Boundary Condition				
				CCCC	CSCS	CECE	EEEE	CFCF
10^8^	10^8^	0	1	8.176	7.378	7.034	6.488	5.551
			2	12.813	10.840	7.711	10.206	5.782
			3	12.813	12.211	12.233	10.206	7.383
			4	14.131	12.385	12.417	11.594	8.896
	10^8^	10^8^	1	8.187	7.390	7.046	6.500	5.560
			2	12.819	10.843	7.711	10.208	5.797
			3	12.819	12.225	12.247	10.208	7.386
			4	14.148	12.391	12.419	11.608	8.913
	10^11^	10^8^	1	9.092	8.179	7.711	7.159	6.302
			2	13.017	10.958	7.883	10.299	6.576
			3	13.017	12.596	12.495	10.299	7.744
			4	15.235	13.050	13.116	12.354	9.783
10^11^	10^8^	0	1	8.176	7.378	7.034	6.488	5.551
			2	12.813	10.840	7.711	10.206	5.782
			3	12.813	12.211	12.233	10.206	7.383
			4	14.131	12.385	12.417	11.594	8.896
	10^8^	10^8^	1	8.177	7.379	7.036	6.490	5.553
			2	12.814	10.840	7.711	10.206	5.784
			3	12.814	12.212	12.233	10.206	7.383
			4	14.132	12.386	12.417	11.595	8.897
	10^11^	10^8^	1	8.199	7.401	7.057	6.511	5.570
			2	12.825	10.846	7.711	10.211	5.810
			3	12.825	12.239	12.262	10.211	7.388
			4	14.164	12.397	12.422	11.622	8.930

## Data Availability

Data are contained within the article.

## Appendix A

$$\begin{array}{l} M=\left[\begin{array}{ccc} M_{UU} & 0 & 0\\ 0 & M_{VV} & 0\\ 0 & 0 & M_{WW} \end{array}\right]\\ M_{UU}=M_{VV}=M_{WW}=\int \int \int \rho H^{T}HdV\\ dV=dxdydz\\ K=\left[\begin{array}{ccc} K_{UU} & K_{UV} & K_{UW}\\ K_{UV}^{T} & K_{VV} & K_{VW}\\ K_{UW}^{T} & K_{VW}^{T} & K_{WW} \end{array}\right]\\ \begin{array}{ll} K_{UU}= & \int \int \int (Q_{11}\frac{\partial H^{T}}{\partial x}\frac{\partial H}{\partial x}+Q_{22}\frac{\partial H^{T}}{\partial y}\frac{\partial H}{\partial y}+Q_{22}\frac{\partial H^{T}}{\partial z}\frac{\partial H}{\partial z})dV\\ & +\int \int \left(k_{x0}^{u}+k_{xa}^{u}\right)H^{T}HdS_{1}+\int \int \left(k_{y0}^{u}+k_{yb}^{u}\right)H^{T}HdS_{2} \end{array}\\ K_{UV}=\int \int \int \left(Q_{12}\frac{\partial H^{T}}{\partial x}\frac{\partial H}{\partial y}+Q_{22}\frac{\partial H^{T}}{\partial y}\frac{\partial H}{\partial x}\right)dV\\ K_{UW}=\int \int \int \left(Q_{12}\frac{\partial H^{T}}{\partial x}\frac{\partial H}{\partial z}+Q_{22}\frac{\partial H^{T}}{\partial z}\frac{\partial H}{\partial x}\right)dV\\ \begin{array}{ll} K_{VV}= & \int \int \int (Q_{22}\frac{\partial H^{T}}{\partial x}\frac{\partial H}{\partial x}+Q_{11}\frac{\partial H^{T}}{\partial y}\frac{\partial H}{\partial y}+Q_{22}\frac{\partial H^{T}}{\partial z}\frac{\partial H}{\partial z})dV\\ & +\int \int \left(k_{x0}^{v}+k_{xa}^{v}\right)H^{T}HdS_{1}+\int \int \left(k_{y0}^{v}+k_{yb}^{v}\right)H^{T}HdS_{2} \end{array}\\ K_{VW}=\int \int \int \left(Q_{12}\frac{\partial H^{T}}{\partial y}\frac{\partial H}{\partial z}+Q_{22}\frac{\partial H^{T}}{\partial z}\frac{\partial H}{\partial y}\right)dV\\ \begin{array}{ll} K_{WW}= & \int \int \int (Q_{22}\frac{\partial H^{T}}{\partial x}\frac{\partial H}{\partial x}+Q_{22}\frac{\partial H^{T}}{\partial y}\frac{\partial H}{\partial y}+Q_{11}\frac{\partial H^{T}}{\partial z}\frac{\partial H}{\partial z})dV\\ & +\int \int \left(k_{x0}^{w}+k_{xa}^{w}\right)H^{T}HdS_{1}+\int \int \left(k_{y0}^{w}+k_{yb}^{w}\right)H^{T}HdS_{2} \end{array}\\ dS_{1}=dydz,\;\;dS_{2}=dxdz \end{array}$$

For Winkler foundation:

$$k_{1}=K_{r},\;\;K_{1}=0,\;\;K_{2}=0$$

For Pasternak foundation:

$$k_{1}=K_{r},\;\;K_{1}=K_{g},\;\;K_{2}=K_{g}$$

For Kerr foundation:

$$k_{1}=\frac{K_{l}K_{u}}{K_{l}+K_{u}},\;\;K_{1}=\frac{K_{s}K_{u}}{K_{l}+K_{u}},\;\;K_{2}=\frac{K_{s}K_{u}}{K_{l}+K_{u}}$$
